# The importance of including occupational therapists as part of the multidisciplinary team in the management of eating disorders: a narrative review incorporating lived experience

**DOI:** 10.1186/s40337-023-00763-6

**Published:** 2023-03-09

**Authors:** Rebekah A. Mack, Caroline E. Stanton, Marissa R. Carney

**Affiliations:** 1 The Johns Hopkins Hospital, 600 N. Wolfe Street, Baltimore, MD 21287 USA; 2 Prisma Health Kidnetics, 29 N. Academy Street, Greenville, SC 29601 USA

**Keywords:** Eating disorders, Occupational therapy, Occupational therapists, Multidisciplinary team, Multidisciplinary care, Identity

## Abstract

The literature demonstrates the importance of utilizing a multidisciplinary approach in the treatment of eating disorders, however there is limited literature identifying the optimal team of professionals for providing comprehensive and effective care. It is widely accepted that the multidisciplinary treatment team should include a physician, a mental health professional, and a dietitian, but there is minimal literature explaining what other professionals should be involved in the medical assessment and management of eating disorders. Additional team members might include a psychiatrist, therapist, social worker, activity therapist, or occupational therapist. Occupational therapists are healthcare professionals who help their clients participate in the daily activities, referred to as occupations, that they have to do, want to do, and enjoy doing. Many factors (e.g., medical, psychological, cognitive, physical) can impact a person’s ability to actively engage in their occupations. When a person has an eating disorder, it is likely that all four of the aforementioned factors will be affected, thus individuals undergoing treatment for an eating disorder benefit from the incorporation of occupational therapy in supporting their recovery journey. This narrative review strives to provide education on the role of the occupational therapist in treating eating disorders and the need for increased inclusion of this profession on the multidisciplinary team. Additionally, this narrative review offers insight into an individual’s personal experience with occupational therapy (i.e., lived experience) during her battle for eating disorder recovery and the unique value that occupational therapy offered her as she learned to manage her eating disorder. Research suggests that occupational therapy should be included in multidisciplinary teams focused on managing eating disorders as it empowers individuals to return to activities that bring personal meaning and identity.

## Abstract (summary)

The recommended approach for individuals participating in treatment for an eating disorder involves the use of a multidisciplinary treatment team which includes a physician, dietitian, and a mental health provider. Sometimes a psychiatrist, social worker, activity therapist, and/or an occupational therapist might also be included in this team. Occupational therapy addresses an individual’s ability to engage in meaningful daily activities. The ability to actively participate in these activities is impacted when an individual has an eating disorder. Therefore, research suggests that eating disorder treatment include occupational therapy to best support the individual in working toward and maintaining recovery, and ultimately empowering them in living life to the fullest.

## Introduction

The literature reflecting the medical assessment and management of eating disorders (EDs) describes the importance of utilizing a multidisciplinary team approach in order to provide the most effective and holistic treatment. Considering the pervasive impact of an eating disorder (ED), multiple practice guidelines as well as other previously published literature recommends the provision of treatment from medical professionals with expertise in various domains to provide the individual with the optimal chance of obtaining a full recovery [1–8]. Despite the consistent message of the need for a multidisciplinary team, there is limited literature depicting which professionals should be a part of that team. Joy, Wilson, & Varechok [6] (pp331) highlight the importance of including various professions when working with individuals who have EDs: “[they] think differently…it is the main reason that multidisciplinary treatment is so valuable. In treating a patient with an eating disorder (ED), no single approach is adequate because the problem itself is multidimensional”. The literature unanimously agrees with the presence of a physician on the multidisciplinary team, albeit there is variation as to whether the physician should be specifically a psychiatrist or any medical doctor (e.g., primary care physician).

The next most commonly recommended professionals to be included in the multidisciplinary team are dietitians and psychotherapists [1, 6]. Then, in no particular order, the following providers are mentioned as potential team members: family therapists, exercise therapists, activity therapists, social workers, nurses, counselors, ED-trained peer support workers, and occupational therapists (OTs) [1, 2, 4, 6, 7, 9]. Notably, these providers are mentioned, but there is not a clear description provided of their roles, outside of the physician (comprehensive evaluation including a medical history, review of systems, physical examination, and laboratory/diagnostic testing), dietitians (assess nutritional status, food attitudes, eating patterns/behaviors), and psychotherapist (screening tools, presenting problem, psychosocial history, mental status, diagnosis, and treatment plan) [6]. One article [6] discusses the physician’s role in asking about the patient’s performance at work, school, and home, as well as their ability to perform self-care tasks; these are areas that an occupational therapist (OT) is highly skilled in assessing and helping the individual to improve.

OTs are healthcare professionals who support individuals experiencing disorders, diseases, illnesses, and injuries through occupational engagement—the participation in meaningful activities of daily living which promote physical and mental wellness [10]. As described in the Occupational Therapy Practice Framework: Domain and Process, OTs work in partnership with clients to analyze the interplay between one’s illness and personal factors (i.e., environment, habits, routines, roles, rituals, values, beliefs) that facilitate or impede a person’s ability to partake in daily activities [10]. OTs describe this as being one’s occupational performance which is “the accomplishment of selected occupation resulting from the dynamic transaction among the client, their contexts, and the occupation” [10] (pp8). Within occupational therapy treatment, a client-centered, collaborative, and goal-oriented approach empowers the individual to increase independence in a broad array of health management skills, thereby facilitating a personal sense of satisfaction and purpose [10]. OTs bring a distinct value to the management of EDs, both within hospital and community-based settings. Yet, OTs are not standard multidisciplinary treatment team members for these illnesses [11]. EDs are serious mental illnesses that lead to pervasive functional deficits in everyday activities, which can be addressed through occupational therapy intervention [11–13]. Through this narrative review, authors describe the role and benefit of including occupational therapy within ED treatment while offering personal insight from an individual with lived experience. Integration of lived experience within research is supported by prior literature, such as that of Beames et al., which states that use of lived experience “supports the identification and development of treatment approaches that align with the needs of those intended to use them” [14] (pp1)]. Consequently, it is important to not only further explore the role and benefit of occupational therapy’s inclusion within ED treatment, but also necessary to determine if this intervention aligns with the needs of those individuals who have ED.

Personal lived experience is provided by M.R. Carney who agreed to collaborate with the primary author (Mack) to discuss her perspective of occupational therapy during ED treatment and the lasting impact on her recovery. Carney worked with Mack in an inpatient hospital setting three years ago, and consequently, it is important to recognize that there is potential for bias by both Carney and Mack. At discharge, Carney initiated a desire to intermittently stay in contact with Mack. Boundaries were put in place by both parties to support professionalism and Carney’s autonomy in her continued recovery. Before, during, and after her time in the Eating Disorder Unit of Johns Hopkins Hospital, Carney has been consistent in her intentions to educate others about the impact of EDs and the importance of asking for and receiving help. For example, Carney shared her lived experience with a group of her teenaged dance students to not only educate them on the importance of addressing mental health but to be a strong role model for them. Additionally, Carney is working on a novella about her anorexia, her experiences of having an ED while undergoing treatment for breast cancer, and parallels between the two illnesses. In being open about her story, Carney aims to impart knowledge and share hope.

## Methods

When the idea for this narrative review originated, Mack asked Carney via email to consider whether she would like to further advocate for ED treatment and recovery by sharing her own personal lived experience. Carney agreed verbally and then written documentation (e.g., ethics approval and consent) was completed to demonstrate Carney’s willingness and desire to be involved in the development of this narrative review. Further ethics approval was unnecessary per Johns Hopkins Hospital’s ethics committee. Mack requested a written response from Carney to the prompt “How did occupational therapy impact your experience within eating disorder treatment and your overall recovery process?” The prompt was intentionally broad and open-ended to prevent bias and allow Carney to share the information she felt was most pertinent to her experience. Carney’s account was written in July 2022, with reflections taken from her personal journals, original poetry and other writings, and artwork during and after treatment. Additional ways that the authors sought to address bias include: Carney was not offered any incentives (e.g., gifts; monetary) to participate; there was no chance that Carney would be provided with any additional treatment or support because she agreed to share her experience; Carney does not have any prior relationship or encounters with Stanton; and Carney was encouraged to leave her personal account unfiltered prior to submission. Carney’s personal lived experience has been edited slightly based on the editorial process.

## Discussion

The profession of occupational therapy is rooted in mental health: it arose as the absence of engagement in meaningful activity was found to be a core issue of the Nineteenth century psychiatric institutions [15]. Unfortunately, few individuals—professional and otherwise—have heard of occupational therapy, let alone understand the vast skill sets it contributes to the client and multidisciplinary treatment team. OTs view any activity that a person engages in as an occupation (i.e., occupational engagement): therefore, OTs help clients work on living life to the fullest by supporting participation (i.e., occupational performance) in the things one needs to do (e.g., responsibilities; hygiene), wants to do (e.g., leisure; hobbies), and enjoys doing (e.g., personal interests) [10].

This description of occupational therapy is reflected within Carney’s personal account:

My favorite part of the day was normally occupational therapy. Besides initially misunderstanding the role of occupational therapy in ED treatment, I also misunderstood that the word “occupational” does not just refer to a job or career. Turns out it includes all the things that make up a person’s day, their environment, and what makes their life meaningful. It had certainly never occurred to me that, much like a stroke survivor, I’d have to relearn aspects of everyday life. Instead of walking or speaking, it was grocery shopping, meal prepping and cooking, and the actual act of eating food. I needed to learn coping skills to eat alone or in social situations. These were just some of the abilities of daily tasks that I lost during my ED.

### Occupational therapy and activity analysis

OTs are trained in the art of activity analysis: examining a task to determine and problem-solve through all elements required to successfully complete it [10]. For example, while preparing a peanut butter and jelly sandwich might seem simple, it is actually quite complex in the number of steps and cognitive processes involved. The person engaging in the activity first must have adequate interoception to identify the body signaling that it needs nutrients and then respond to this signal by initiating the food preparation task. Next, the person must have the necessary executive function skills to sequence a multi-step activity. The person must have appropriate grip strength, range of motion, and visual perception to locate and then obtain the necessary materials. The person’s distal control, fine motor coordination, force gradation, and strength must support them in spreading the peanut butter and jelly onto the bread with minimal mess. The person needs to be able to problem-solve how much peanut butter and jelly to spread on the bread and when to terminate the task. The person must have the sensory processing skills to modulate the visual, tactile, and olfactory stimuli experienced during this task. Already, a multitude of steps have been identified that are needed to make preparing a sandwich a successful experience, and the task is not yet finished. This knowledge and understanding of daily occupations, the associated cognitive processes, and the meaning ascertained to each individual’s occupations reflect an OT’s expertise.

### Impact of ED on occupational performance

EDs have a high mortality rate [1, 16, 17], and pervasively impact one’s life [18]. An ED at minimum will affect the way that an individual interacts with food, engages in social situations involving food (e.g., parties; holidays), manages their roles (e.g., family member, employee), and perceives themselves (i.e., identity, self-esteem) [11–13, 19, 20, 22, 26]. The literature documents that additional areas impacted include but are not limited to: hygiene; school/work involvement; ability to care for others; physical health; mental/emotional health (e.g., mood, anxiety); finances; socialization (i.e., isolation); relationships; communication via increased secrecy; ability to cope positively; emotional regulation; and ability to contribute to society [2, 11, 12, 19–26]. All of the aforementioned aspects are areas an OT is skilled in addressing, ultimately supporting the individual in living their life to the fullest. In doing so, OTs adopt a holistic approach by treating areas including, but not limited to:

- "Appropriate eating behaviors
- Client factors specifically related to triggers to engaging in ED behavior, warning signs of relapse, and positive coping strategies
- Balanced occupational engagement
- Desired role performance, in contrast to illness role performance
- Emotional regulation
- Self-esteem
- Environmental and contextual implications
- Impaired functional cognition related to malnutrition
- Performance patterns
- Interpersonal skills
- Overall health management” [13] (pp17).

Participating in daily activities that an individual derives personal meaning from fosters the belief that one’s life is valuable, aids in the formation of identity, and promotes feelings of satisfaction, competence, and belonging [27, 28]. With EDs, these typical daily activities—such as eating, cooking, shopping, exercising, socializing, and working—evolve around the disordered cognitions and are executed in a manner that sustains the ED and hinders one’s physical and mental health [12]. For example, participation in exercise is often driven by an intense urge to burn calories to promote feelings of control over one’s body shape and size, even when an individual is already medically compromised due to malnourishment and/or being at a low body weight [11]. Grocery shopping and meal preparation may provoke dysregulated emotional states and involve rigid routines [11]. Self-care activities such as bathing and dressing may be challenging due to exposure to one’s own physical body or physical weakness [11]. A person’s daily routine oftentimes revolves around these time-consuming disordered behaviors and cognitions, leading to an occupational imbalance in which one does not engage in meaningful and health-promoting activities.

The impact of an ED on one’s occupational engagement is evident throughout Carney’s personal account:

My anorexia included severe restriction and over exercise. It took control of me very quickly, and I couldn’t get out of it on my own.

I work in media relations for a Big 10 university. While that includes a bunch of different facets, most of my job is writing. The deeper into anorexia I slid, the harder that became. The brain fog was real. I couldn’t focus, couldn’t organize my thoughts, couldn’t write more than a few (crappy) sentences at a time. An article that I once could have written in a day or two started taking a week or longer. I was frustrated because I didn’t really understand what was happening to me, but I was more apathetic about it. I’m a hustler, baby, so this was all totally out of character.

I’m also a dance instructor and was teaching two nights a week when things started to get really bad. I’m a very hands-on, loud, engaged teacher. I’m talking full-out demonstrations, full-out dancing alongside the students, much cheerful yelling, lots of emphatic counting and clapping and various unintelligible noises. As the weight sloughed off and my level of nourishment plummeted, I couldn’t keep up with my normal style of teaching. I was winded after a half-hearted attempt at a demonstration. I barely had the energy to speak, let alone holler over the music. I had to cut back on my classes the year before I went to treatment. Gone was my vibrant personality. My students were paying a price, and that wasn’t fair to them. I don’t teach because I need the money—dance is my passion, my expression, where I feel like I can make an impact. So I knew I should be upset about it, but I was too numbed out, too depleted to be anything but indifferent.

The two biggest portions of my life were suffering terribly and so were the many other smaller pieces. I am a voracious reader, but I couldn’t focus or absorb what was on the pages anymore. I love being in nature and doing physical activities, but I was too tired, and eventually too weak, to hike or play sports. I had no interest in even spending time with my nieces and nephew. I avoided family events or hanging out with friends because I didn’t want to be around food or fake my way through conversations.

Anorexia was taking everything from me. I knew if I didn’t get into treatment, it would take my life, as well.

### Occupational therapy treatment

OTs support individuals in learning how to engage in these daily activities while managing the impact of the ED on one’s participation. In doing so, OTs implement various techniques such as activity analysis, environmental adaptation, and positive functional coping skills to manage distress and support occupational performance [11, 19, 20]. For example, an OT may work with a client on their ability to plan, grocery shop for, prepare, and consume a meal that adheres to their nutritional needs and meal plan requirements. While doing so, the OT and client work together to challenge any disordered thoughts or behaviors the client has while engaging in the task. This may include a multitude of actions such as selecting a meal that does not conform to the individual’s rules related to foods they will/will not consume, walking a different route in the grocery store, purchasing items without reading nutritional labels, choosing a different brand of a food item, using less rigid food preparation methods, consuming the prepared meal, and deferring from using disordered or compensatory behaviors (i.e., bingeing, purging, restricting). The OT works with the client throughout this process to reframe disordered cognitions and utilize positive coping skills [20]. By implementing activity-based interventions within the contextual environment and providing graded support, OTs support the client to help them succeed in taking action steps that promote recovery [11]. Attaining success leads to improved self-esteem and a gradual increase in one’s independence in completing the task [11, 13]. OTs’ expertise and ability to analyze the skills a client requires to complete a task and to provide graded support are a unique contribution to the client and multidisciplinary treatment team.

Engaging in disordered eating behaviors becomes what gives an individual meaning, value, and purpose in life [12, 21, 27]. This occurs because EDs alter the mechanism by which individuals derive meaning via changes in the dopamine reward system, which alter one’s mediation of pleasure [29, 30]. Following the rules created by the disorder (i.e., calorie limitations, food restrictions, use of compensatory behaviors) provides the individual with a sense of control, structure, and routine. Attaining disordered goals (i.e., weight loss, physical appearance goals) fosters a sense of accomplishment (i.e., mastery) and achieved perfection. Individuals with EDs may become known by others for their ability to demonstrate restraint while eating, their dedication to exercise, and their ability to control their body size/shape, thereby leading to a sense of pride and further development of an identity based in their disorder [12]. The ED serves as a source of comfort, solace, and protection: it is a coping mechanism, yet it can lead to physical and psychiatric morbidity and mortality [16–21, 27]. As described by Lavis [21] (pp457), the ED can “be a paradoxical mode of self-care”. The recovery process is often associated with distress related to the inability to attain this self-care through disordered behavior [21]. Thus, the development of positive coping skills, engagement in supportive self-care, and formation of identity outside of the ED are essential to recovery.

OTs assist clients in discovering their identity outside of the ED. OTs challenge the assumption that the individual is defined by their disorder by collaborating with them to analyze their personal beliefs and values. OTs also promote formation of identify by supporting clients in successful engagement in activities that promote a sense of purpose and meaning in life [12, 27, 31]. These activities may be skills or hobbies such as art, reading, socialization, or academics. In some cases, individuals may need support in re-engaging in activities they previously enjoyed but have become apathetic toward. In other cases, clients may need assistance in ascribing new meaning to the activity; for example, mindful movement for leisure rather than obsessional exercise [11, 12]. OTs also work with clients to identify the roles they want and need to fulfill: for example, sibling, parent, employee, student, caretaker of animals, friend [32]. Envisioning being able to fulfill a meaningful role without the restraints of the disorder oftentimes serves as a motivating factor in the pursuit of recovery. OTs collaborate with clients to plan and implement action steps to resume or improve performance in these roles and activities [32]. A study by Dark & Carter [33] showed that despite occupational engagement increasing recovery from an ED, participants identified that greater transformation occurred when the meanings associated with occupations changed. Forming one’s purpose apart from the ED is an essential step in promoting recovery [12], as it empowers individuals to participate in daily life without relying on the disorder to sustain their identity.

When working in higher levels of care, OTs aid clients in developing skills necessary for continuing to work towards recovery as they transition to lower levels of care. During this transition, clients are vulnerable to relapse due to the vast array of challenges they encounter when reintegrating into the community [34]. Some of these challenges include managing the loss of the structure and support provided through intensive treatment, scheduling and planning for meals, identifying purpose outside of the ED, coping with triggers (i.e., media, comments from others), and resuming school/work/family roles [34]. OTs collaborate with the client to identify and problem-solve strategies to manage these challenges and pursue recovery. Literature states that techniques such as time management, assertive communication, engagement in leisure activity, emotional expression, seeking support from others, and positive coping skills promote recovery [34]. These are all skills that an OT can teach to a client while providing graded support as the client learns to implement them with decreased assistance. Therefore, it is suggested that OT’s have an essential role in supporting individuals as they learn to re-engage in the world outside of their ED.

Carney provides insight into her experience with inpatient occupational therapy treatment through her personal account:

In occupational therapy, it wasn’t just that the OT was always prepared and kind and compassionate toward us. Or that we played fun games to start off the session. Or that I felt a tiny bit less pressure to be perfect there. I got a lot out of occupational therapy because we used writing, drawing, and other forms of art to express ourselves and reinforce the main points of what we were covering that day. We made a lot of lists, and damn do I love making lists. Our lists had lists. We made goals and talked about how we could attain them. We shared barriers to our recoveries and came up with ways to overcome them. We dug into motivations for recovery. We talked about attainable actions and the results/benefits of recovery.

Eventually, I was able to participate in Food Prep Fridays, which is just what it sounds like. We’d plan a menu, prepare, then eat the food we made according to our personal meal plans. Honestly, the only thing I liked about these Friday lunches was getting off the unit for a few hours.

Holy hell did I loathe everything else about it. I hate cooking, I always have. No part of it is fun for me. I don’t like the smell of cooking food. I never have, even before the ED. I hated that we were not allowed to clean up as we went. Messes are also not fun for me. And I like efficiency. It may have been a little bit fun to plan for some different foods than we ate on-unit, but actually having to look at it, make it, and eat it was another story. It was not unusual for me to cry before, during, or after the meal. More often, it was all three.

But I knew it was another important step in preparing me to transition to the day hospital where I’d be reintroduced to eating out in restaurants and become increasingly responsible for feeding myself. Let me be clear: nothing made any of it easier or less painful. Nothing. But the practice, the discussions, the discoveries from occupational therapy and groups were fresh in my mind to reach for when I needed them.

As I got closer to my discharge, the OTs worked with me to create some meal plan ideas and options, lay out a support system of family, friends, and professionals, and put in place relapse prevention plans.

All to say that with occupational therapy, it felt like we were doing tangible, physical things to keep us working hard in the hospital, but that laid a recovery foundation for once we returned home.

### Summary of personal account

Carney’s reflections on her experience with an ED reveal that she was impacted mentally, emotionally, neurologically, and physiologically. She became entrenched in new habits and routines specifically related to the ED behaviors of restriction and over exercise. Carney shared how her role as a writer was significantly impacted, as it became difficult for her to focus, articulate her thoughts, and write effectively. Completing previously "simple" tasks now required significantly more time. Carney’s drive to do well and take pride in her work was replaced with apathy—a common experience for individuals struggling with EDs. The ED continued to take over her life, and also stole from her identity: where she had previously been a very engaged and interactive dance teacher, Carney now physically, mentally, and emotionally could not maintain: “gone was my vibrant personality”. Emotionally, Carney verbalized that she knew that feeling upset would be rational and logical, but the ED had sucked so much from her that she instead felt nothing—“numb… [and] indifferent”. Carney’s ability to engage in meaningful leisure activities was impacted. She also became more isolated and withdrawn from the people she loved, thus impacting her socialization.

Carney shared that her experience with occupational therapy during treatment for her ED focused on addressing “all the things that make up a person’s day, their environment, and what makes their life meaningful.” She highlighted the focus on grocery shopping, meal preparation, cooking, eating food, positive coping skills, and socializing with others. Carney also emphasized occupational therapy’s intentionality with setting goals, identifying barriers, and then problem-solving strategies to support goal completion. She reflected on how the foundational work in occupational therapy ultimately supported her when she stepped down to a lower level of care (day hospital), especially related to developing meal plans and relapse prevention plans.

Carney summarizes the importance of occupational therapy for her recovery process with the following:

Without occupational therapy I know that I would not have learned or come to understand as much about my anorexia. I know that I would have been missing critical skills to stay the course, especially over the next two years.

I was discharged from Johns Hopkins Hospital in July 2019. By the following March, the COVID pandemic was raging. My university shut down, and I was working from home. My dance studio closed, and I was conducting classes over Zoom. No visits from family or friends. Eating disorders love isolation. Grocery store shelves were bare. Eating disorders thrive on food anxiety.

In April, I was diagnosed with breast cancer. In a world of isolation. With an eating disorder.

I had to really dig deep for my strength to stay the recovery course. I had to use every single tool I’d been given, every coping technique I’d been taught to get through my situation, many of which I’d learned in OT. I leaned into those things like reframing and distraction. Most helpful was the meal planning and prep exercises that I worked hard to keep implementing daily. I even recreated a few activities at various times. One was drawing a tree from roots to trunk, limbs, and leaves, and labeling each with things like fears, strengths, supports, and hopes. Another was a recovery collage/vision board.

I firmly believe occupational therapy has a place in ED recovery. I also know there is room for it to grow, so much more it could include for the benefit of those suffering. When this is understood, occupational therapy can be more widely accepted, funded, and given its proper place in treatment programs.

## Conclusions

EDs are complex illnesses which multifariously impact one’s well-being, therefore provision of comprehensive treatment is essential. The inclusion of occupational therapy services bolsters the comprehensive nature of treatment as OTs possess a unique skill set in promoting management of EDs through multi-faceted approaches, including behavioral, psychosocial, environmental, cognitive, medical, and interpersonal. OTs are uniquely skilled in identifying the meaning that provokes participation in an activity and understanding how this engagement impacts one’s well-being and sense of self (i.e., identity) [12, 27]. The multidisciplinary team commonly involved in treating EDs includes a physician, a mental health provider, and a dietitian [35]. These professionals are crucial in supporting an individual’s recovery from an ED; however, none are experts in addressing meaningful occupational engagement [11]. Engaging in daily life activities in a health-promoting manner is essential to enabling recovery, thus OTs are an essential member of the multidisciplinary team for the assessment and management of EDs.

## Data Availability

Not applicable.

## Abbreviations

ED: Eating disorder
EDs: Eating disorders
OT: Occupational therapist
OTs: Occupational therapists
